# Strains of *Aureobasidium pullulans* from Extreme Environments: New Potential Biocontrol Agents?

**DOI:** 10.3390/microorganisms13112596

**Published:** 2025-11-14

**Authors:** Martina Lucci, Nataliia Khomutovska, Giuseppe Firrao, Alessandra Di Francesco

**Affiliations:** 1 Department of Agricultural, Food, Environmental and Animal Sciences, University of Udine, 33100 Udine, Italy; lucci.martina@spes.uniud.it (M.L.); giuseppe.firrao@uniud.it (G.F.); 2 Department of Plant Protection Biology, Swedish University of Agricultural Sciences, 234 22 Lomma, Sweden; nataliia.khomutovska@slu.se

**Keywords:** cold environment, desert, urban sites, extremophiles, postharvest management

## Abstract

Extreme environments are a largely unexplored reservoir of microbial diversity, with a remarkable potential to be exploited in agriculture. One hundred and seventeen yeast isolates, derived from different ecosystems in Italy, Sweden, Algeria, and France, were molecularly identified, and the most represented genus was *Aureobasidium* (57%). A phylogenetic analysis based on a multi-*locus* sequence typing (ITS, ELO, EF-1alpha) was conducted to characterize the black yeasts’ population. To investigate *A. pullulans* extremophilic and extremotolerant behaviour, different temperatures and pH, together with the enzymatic production, were evaluated. The strains were tested by in vitro and in vivo assays against the postharvest fungal pathogen *Monilinia fructicola* as potential biocontrol agents (BCAs). Results displayed a great ecological variability concerning strains’ growth and cell production depending on different culture conditions. However, a remarkable thermotolerance aptitude was detected in almost all the strains. In particular, the strains belonging to Group 2 (Algerian Desert) and 3 (Alto Adige Region) showed, respectively, higher thermotolerance and biocontrol ability. These findings showed how some extreme environments could represent a promising source for new potential BCAs. However, further studies are needed to investigate the mechanisms of action of these putative BCAs for application during the postharvest phase.

## 1. Introduction

Nowadays, climate change and the resulting degradation of the environment are major issues that need to be addressed and managed [1]. The multiple causes of this crisis are primarily linked to human activities that increased greenhouse gas levels, leading to global warming and changes in weather patterns [2]. Extremophilic microorganisms may represent some of the oldest life forms on our planet, demonstrating nature’s resilience at extreme salinity, pH, and temperature [3]. These microorganisms display a unique genetic adaptation that allows them to thrive where other microorganisms often do not. As largely unknown entities, they have attracted the scientific community’s attention mainly for their ability to produce bioactive compounds [4]. Their specialized adaptation not only enables them to survive in harsh environments but also paves the way for environmentally friendly and efficient alternatives to the use of agrochemicals in agriculture, food production, cosmetics, and pharmaceuticals [4,5].

In the case of yeasts, they are particularly able to survive in a wide range of biomes, including extreme conditions defined by cold, heat, drought, acidity, alkalinity, salinity, osmolarity, toxicity, and UV exposure, alone or in combination. Different yeast species, ascomycetes and basidiomycetes, were detected in extreme environments and recognized as being well-suited to extreme conditions [6,7,8,9,10]. Among the black yeasts, an ecological group of melanized microorganisms considered to be the most stress-resistant eukaryotes, *Aureobasidium pullulans* is the most abundant species [11]. *Aureobasidium pullulans* is a poly-extremotolerant microorganism, epiphyte or endophyte, globally distributed from tropical to polar areas [12,13]. It is also recognized for its role as a biocontrol agent (BCA) of the most detrimental pathogens of fruits in the postharvest phase [14,15,16], showing its potential as a sustainable alternative to synthetic fungicides [17]. In particular, a significant number of pesticides are presently used to prevent brown rot on stone fruits, caused by *Monilinia* spp., which are pathogens that produce severe losses in worldwide stone fruit production, with high economic relevance (1.7 million EUR/year) [18]. Among the known species, *Monilinia fructicola* is one of the most widespread diseases of stone fruits [19,20]. *Aureobasidium pullulans* is an extremotolerant generalist that can withstand a variety of extreme conditions. It is often ubiquitous, nutritionally versatile, and easy to cultivate, making it difficult to outcompete [21]. Black yeast is able to increase intracellular and decrease extracellular glycerol concentrations at high salinity conditions or maintain high fluidity of the plasma membrane, important parameters of stress tolerance [22,23].

In the present study, 67 strains of *A. pullulans* were isolated from different environments characterized by extreme conditions, with the aim to (i) evaluate their growth on different media, temperatures, and pH values; (ii) verify their production of cell wall degrading enzymes (CWDEs) and siderophores; and (iii) assay by in vitro and in vivo experiments their potential as BCAs against *Monilinia fructicola* of stone fruit.

## 2. Materials and Methods

### 2.1. Sampling and Strain Isolation

During winter 2023 and early spring 2024, 117 microorganisms were isolated from samples collected in different environments such as urban centres, deserts, lakes, coasts, and woods (Supplemental Material Figure S1). Rocks, sand, moss, grass, leaves, mushrooms, snow, ice, and water were the sampled materials. Solid samples (4 g) were washed with 20 mL of sterile distilled water (SDW) amended with Tween 20 (0.05% *v*/*v*, Sigma-Aldrich, St. Louis, MO, USA), collected in sterile flasks and subsequently placed at 20 °C for 1.5 h under continuous agitation on a rotary shaker (250 rpm). Samples’ washing water was centrifuged for 1 h at 4000 rpm, and then the supernatant was discarded. The pellet was suspended with 1 mL of SDW, and then 100 μL of each solution was spread on Petri dishes containing NYDA (8 g L^−1^ nutrient broth, 5 g L^−1^ yeast extract, 10 g L^−1^ Dextrose, and 25 g L^−1^ of Agar Technical). Liquid samples (40 mL) were filtered through a 0.2 μm-pore-size Sterivex filter unit (small fraction; Millipore, Burlington, MA, USA), and each filter was placed on a Petri dish containing NYDA media, following the method of Kutty (2009) [24]) with some modifications. In both cases, plates were incubated at 20 °C for different times, from 48 h to 240 h. Single colonies displaying yeast morphological characteristics at the optical microscope (Zeiss AXIO Observer.Z1, Jena, Germany) were selected and streaked on new plates with a sterile loop. Cultures were later purified and stored at −80 °C until use.

### 2.2. Pathogen and Fruit

*Monilinia fructicola* strain AMF1, belonging to the mycological collection of Di4A-Department of Agricultural Sciences of Udine University, was used as a target pathogen to test the potential biocontrol activity of the characterized yeasts. The fungal pathogen was grown on PDA (Potato Dextrose Agar, Oxoid, Basingstoke, UK) for 5 d at 20 °C before use. Peaches (*Prunus persica* (L.) Batsch) *cv* “Red Haven” were harvested at commercial maturity (10 °brix) in an organic orchard located in San Vito al Tagliamento (Pordenone, Italy) and stored at 4 °C until use. Fruits homogeneous in size and with no signs of damage were selected.

### 2.3. DNA Extraction and Analysis

The yeast isolates were cultured in flasks containing NYDB medium (NYDA without agar). The cellular cultures were centrifuged at 3500 rpm for 30 min, and the supernatant was discarded. The cells were then frozen using liquid nitrogen and stored at −80 °C. Genomic DNA was extracted using 150 mg of each sample, collected in 2 mL microcentrifuge tubes, following the protocol described by [25]. To characterize the cultures, the Internal Transcribed Spacer (ITS) nucleotide region was initially considered. Later, a multi-*locus* approach was considered to characterize *Aureobasidium* strains through the translation elongation factor EF-1α gene (EF1) and part of the elongase gene (ELO). The ITS region was PCR-amplified using the primers ITS1 (5′-TCCGTAGGTGAACCTGCGG-3′) and ITS4 (5′-TCCTCCGCTTATTATTGATATGC-3′) as described by [26]. For amplification of the elongase gene, primers ELO2-F (5′-CACTCTTGACCGTCCCTTCGG-3′) and ELO2-R (5′-GCGGTGATGTACTTCTTCCACCAG-3′) were used, following the protocol of Zalar et al. (2008) [27]. The elongation factor 1α gene was amplified using the primers EF1-728F (5′-CATCGAGAAGTTCGAAGG-3′) and EF1-986R (5′-TACTTGAAGGAACCTTTACC-3′), as reported by [28].

PCR amplifications were carried out separately on a MiniAmp Plus thermal cycler (Thermo Fisher Scientific, Waltham, MA, USA). For the ITS region, an initial denaturation of 94 °C for 2 min was followed by 40 cycles of 94 °C for 40 s, 55 °C for 40 s, and 72 °C for 1 min, with a final extension of 5 min at 72 °C. For ELO and EF1 regions, the parameters were as follows: initial denaturation at 94 °C for 5 min, 35 cycles of 94 °C for 15 s, 56 °C for 40 s, and 40 s at 72 °C, with a final extension of 8 min at 72 °C [16]. PCR products were purified and sequenced by BMR Genomics (Padova, Italy). The ITS, EF1, and ELO sequences of *Aureobasidium* spp. were used to build a concatenated phylogenetic tree by using Seaview 5.0.5 [29].

### 2.4. Aureobasidium pullulans Ecological Studies

Sixty-seven *A. pullulans* strains were evaluated for their ability to grow on different media at different temperatures and pH conditions. Regarding the media, Czapek Dox Agar (49 g per 1 L of SDW) (Oxoid, UK) and NYDA, respectively, a minimal and a nutrient medium were used. Ten mL of each medium were poured into each well of the 6-well cell culture plates (Sarstedt, Nümbrecht, Germany) and later inoculated with a plug (4 mm diameter) of each strain taken from the margin of active growing colonies. Plates were incubated at five different temperatures ranging from 0 °C to 45 °C. Colony growth and conidial production were measured 14 d post inoculation (dpi) at 10 and 25 °C, 21 dpi at 0 °C and 5 °C, respectively. Two different timings of colony diameter growth detection were chosen in order to standardize the development of colonies that had not grown after two weeks at 0 °C and 5 °C of incubation.

After 7 d at 45 °C, where the colonies showed no growth, plates were incubated for another 7 d at 25 °C to verify the strains’ tolerance to low and high temperatures, respectively. Colony diameter was measured at two perpendicular axes. To determine cell production by each strain at each condition, plates were filled with 2 mL of SDW and scraped with a sterile loop. Strain cell suspensions were collected in sterile tubes (2 mL), and the concentration was determined by a Thoma cell. A sample unit for each isolate, medium, and temperature was represented by 3 wells of each plate.

To evaluate the strains growth ability at different pH conditions, Czapek Dox Agar medium was adjusted to 2, 4, 6, 8, 10, and 12 pH. Plates were incubated at 25 °C for 12 d. The colony growth and cells production were measured as reported above. A sample unit for each isolate and pH was represented by three wells of each plate. The experiments were conducted once.

### 2.5. Dual Culture Assay

To evaluate the efficacy of all 67 *A. pullulans* strains as BCAs, a co-culture assay with *M. fructicola* strain AMF1 was performed. On PDA plates (90 mm diameter), a mycelial plug of the pathogen (6 mm Ø) collected from a 7 d-old colony was placed 25 mm from one edge of the plate, and a loop of yeast cells derived from a 2 d-old culture was streaked on the other side of the plate at the same distance, as reported by [17]. Plates were incubated at 25 °C for 4 d. This temperature was selected as it is optimal for the growth of *A. pullulans*. The sample unit of each assay consisted of three plates for each interaction. Plates inoculated only with the pathogen represented the control. The experiment was conducted twice. The inhibition rate of the mycelial growth was calculated using the following formula:

% Inhibition = (d1 − d2)/(d1) ∗ 100

where d1 and d2 are the control colony and the treated colony diameters, respectively [30].

### 2.6. Aureobasidium pullulans Efficacy Against M. fructicola on Peaches

Peaches *cv* “Redhaven” were disinfected by immersion for 1 min in sodium hypochlorite solution (1%) and suddenly washed twice with tap water [16]. After drying, fruits were wounded (2 × 2 × 2 mm) three times with a sterile needle. Each wound was inoculated with 15 μL of each *A. pullulans* strain suspension (1 × 10^8^ cells mL^−1^). After complete adsorption (1 h), 15 μL of conidial suspension (1 × 10^5^ conidia mL^−1^) of the pathogen was pipetted into the same wounds. Fruits were then incubated for 7 d at 20 °C. The percentage of infected wounds and the disease severity (mm) were measured. Fruit inoculated with SDW (15 μL) represented the control. The sample unit consisted of five fruits, and the experiment was conducted twice.

### 2.7. Cell Wall Degrading Enzymes (CWDEs) and Siderophores Production

Twelve strains of *A. pullulans* were selected, based on their different ability to inhibit *M. fructicola* by in vitro and in vivo assays, to evaluate their CWDEs and siderophore production (Table 1). The cellulase and xylanase activity, as well as the production of siderophores by the selected strains, were evaluated. Cellulase (endo-1,4-*β*-glucanase) and xylanase assays were performed. For cellulase activity, agar medium consisted of PYE (peptone 0.5 g; yeast extract 0.1 g; agar 16 g L^−1^ for 1 L of SDW) supplemented with 0.5% Na-carboxymethylcellulose (Sigma-Aldrich, St. Louis, MO, USA) [31]. For the xylanase, the agar medium was prepared by using 0.5% beech wood xylan (Sigma-Aldrich), NaNO_3_ 0.3%; KH_2_PO_4_ 0.1%; MgSO_4_ 0.05%; yeast extract 0.1%; agar 1.2% for 1 L of SDW [32]. The substrates were poured into 90 mm-diameter Petri dishes and, after solidifying, were drilled with a sterile cork-borer (6 mm Ø). Each empty well was filled with 50 µL of each yeast strain’s suspension (1 × 10^8^ cells mL^−1^). After 24 h at 25 °C, the enzymatic halos produced by each strain were visualized only after the staining of the plates with 5 mL of a 0.2% Congo Red solution for 15 min, after discolouring with the same amount of NaCl (1 M). The diameter of the enzymatic halos was measured by a digital caliper. For each strain and enzyme, five plates were prepared.

The siderophore assay was assessed following the protocol reported by [33,34]. The Petri plate was composed by NYDA on one half and an agar medium supplemented with chrome azurol S (CAS) and 1,4-piperazinediethanesulfonic acid (PIPES) (Sigma-Aldrich) on the other. In the centre of each plate, a plug (4 mm Ø) of 48 h yeast cells was inoculated. Plates were incubated at 25 °C for 1 month in the dark. For each strain, three plates were prepared. All the assays were conducted twice.

### 2.8. Data Analysis

All the data were subjected to one-way analysis of variance (ANOVA). The statistical comparisons of means were established with Tukey’s HSD Test (α = 0.05) by using Minitab 17^®^ statistical software (Minitab, State College, PA, USA). Correlations among variables were calculated and plotted using Rstudio [35]. Non-metric multidimensional scaling (NMDS) was conducted in RStudio to evaluate the similarity and dissimilarity among *A. pullulans* strains. NMDS was performed using the “metaMDS” function from the “vegan” package [36], which applies Bray–Curtis dissimilarity and stress-based minimization to represent multidimensional trait data in two dimensions. The supervised machine learning method was used to assess the relative importance of phenotypic variables in predicting the group of origin using NMDS. Variable importance was evaluated based on the increase in node purity (IncNodePurity), with results visualized using “ggplot2” [37] to highlight the most relevant conditions associated with strain groups.

## 3. Results

### 3.1. Molecular Characterization of Extremophile A. pullulans Strains

**Figure 1:**
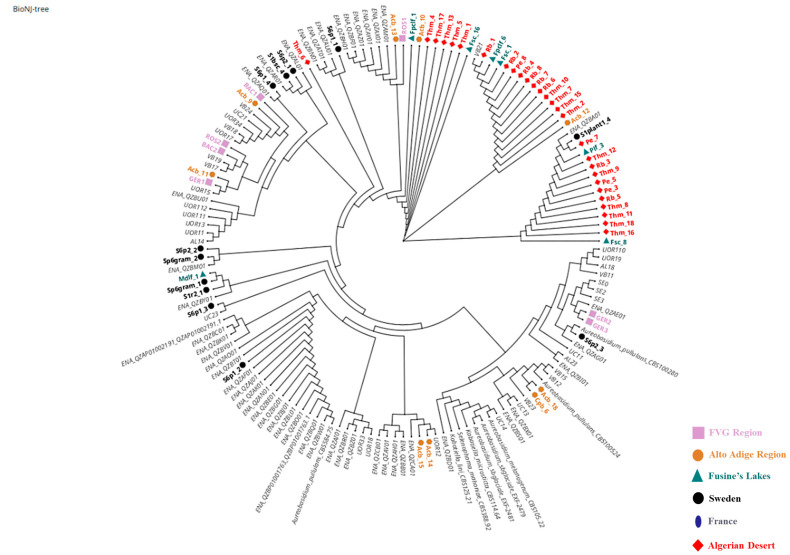
Phylogenetic analysis of *Aureobasidium pullulans* isolates. Graph built using Neighbour-Net method based on the concatenated ITS + ELO + EF1 genes. The strains from FVG Region (Group 1) are marked by a pink square (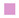), from Algerian Desert (Group 2) with a red rhombus (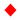), from Alto Adige Region (Group 3) with an orange circle (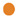), from Sweden coast (Group 4) with a black circle (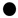), from Fusine’s Lake (Group 5) with a blue triangle (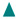), and from France Urban Centre with a blue ellipse (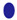). The reference sequences are reported with a light grey colour. The light grey reference sequences were retrieved from the European Nucleotide Archive (ENA) or were obtained by Cignola et al. 2023 [16] and courteously provided by the authors for this figure.

One hundred and seventeen colonies were morphologically identified by optical microscope as yeasts. All the purified cultures were subsequently analyzed using molecular methods. The amplification of the ITS gene that showed a sequence of 508 nucleotides corresponded to *Aureobasidium* spp. for 67 isolates. The other 50 strains were represented by *Cryptococcus* (12%), *Vishniacozyma* (7%), and *Naganishia* (6%) (data not reported). *Aureobasidium* strains were suddenly analyzed with two target genes, ELO and EF-1alpha, displaying sequences of 690 and 230 bp, respectively. Thanks to the variability of these *loci*, more discriminant than the ITS, all the 67 strains were identified as *A. pullulans* (Table 2). The 67 *A. pullulans* strains were brought together in six groups, each related to the geographical origin: Friuli Venezia Giulia (FVG) (Group 1), Algerian Desert (Group 2), Alto Adige Region (Group 3), Sweden Coast (Group 4), Fusine’s Lake (Group 5), and France Urban Centre (Group 6). A phylogenetic tree was constructed using the concatenated alignment of ITS, ELO, and EF-1alpha *loci* (Figure 1). As shown in Figure 1, all the strains were grouped by sampling location. A corresponding colour and symbol were assigned and highlighted for each geographical origin in the legend. The strains from the Algerian Desert were almost all included in two clades with respect to the rest of the population that displayed a heterogeneous distribution, forming much smaller clusters, most of which were very close to the reference sequences deposited on GenBank^®^.

### 3.2. Aureobasidium pullulans Ecological Study: Colony Growth

All the *A. pullulans* strains were grown on different media, including NYDA and Czapek Dox agar at 0 °C, 5 °C, 10 °C, 25 °C, and 45 °C, respectively. Colony mycelial growth and conidial production were evaluated after 2 weeks (10 °C and 25 °C), 3 weeks (0 °C and 5 °C), and 1 week (45 °C) and are reported as averaged data for each group (Table 3). For each group, only a few strains were thermotolerant to 45 °C, except for Group 4 (Sweden coast), in which any strains showed ability to grown at 45 °C on NYDA and Czapek agar (data not reported). Conversely, all the strains grown on NYDA and Czapeck agar resist 0 °C, showing large differences among strain groups and media. The colony diameters ranged from 2.04 mm (Group 5) to 5.58 mm (Group 6) on NYDA and from 2.23 mm (Group 5) to 5.57 mm (Group 1) on Czapeck. The largest colony diameters were measured at 10 °C and 25 °C. At 10 °C, Group 2 (Algerian Desert) and 6 (France Urban Centre) showed colony diameters of 21.21 mm, 24.92, 21.04 mm, and 24.0 mm, respectively. Between NYDA and Czapeck agar, statistically significant differences were found. At 25 °C, Group 6 on Czapeck showed the largest colony diameter (27.25 mm). Results showed that Groups 1 and 6 demonstrated a higher ability to grow at different temperatures and media. Generally, Group 3 (Alto Adige Region) and 4 (Sweden Coast) displayed the lowest colony diameter at the different tested temperatures.

### 3.3. Aureobasidium pullulans Ecological Study: Cells Production

After the incubation time at different temperatures, cell production (cells/mL) was measured for all the strains clustered in the six different groups. As reported in Table 4, temperature and medium strongly influenced the yeasts’ cell production (cells/mL). In effect, Czapeck medium slightly reduced cell production of the strains clustered in the six groups when compared to NYDA medium. At the same time, Groups 4 (Sweden Coast) and 6 (France Urban Centre) showed the highest number of cells per mL independently from the temperature of incubation on NYDA.

### 3.4. Influence of Different pH Values on Aureobasidium pullulans Colony Growth

*Aureobasidium pullulans* colony diameter (mm) was measured on NYDA plates at six different pH values (from 2 to 12) (Supplemental Material Figure S2). Table 5 showed how all the isolates clustered in the six groups were able to grow in a wide range of pH after 12 d of incubation at 25 °C. Isolates belonging to Groups 2 (Algerian Desert) and 6 (France Urban Centre) displayed the largest colony diameters, especially at pH 12, reaching 26.98 mm and 28.63 mm, respectively. Isolates clustered in Group 1 (FVG Region) grew better with lower pH values, from 6 to 2. Conversely, Groups 5 (Fusine’s Lake) and 3 (Alto Adige Region) showed good growth ability only at pH values ranging between 6 and 8, showing a significant reduction at more extreme pH conditions (pH 12, 10, 4, 2).

### 3.5. Dual Culture Assay

**Figure 2:**
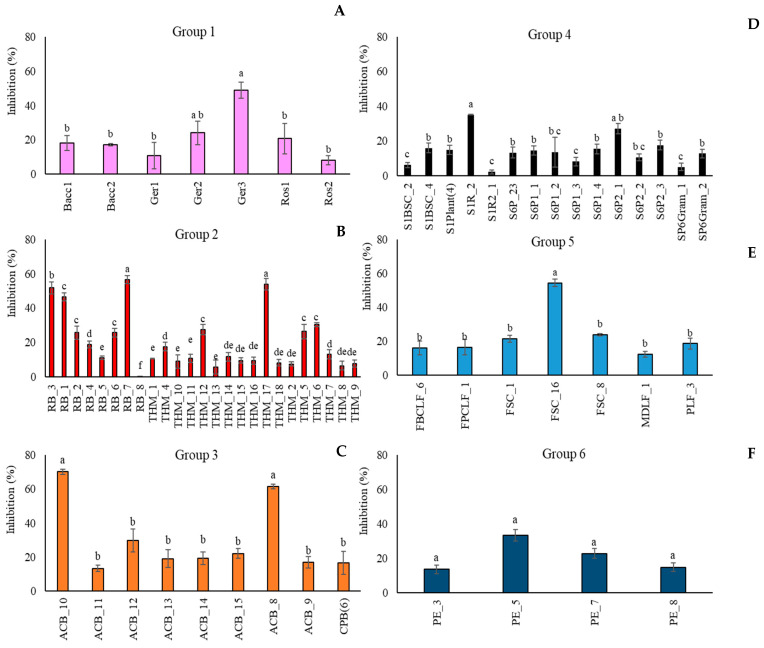
Percentage of inhibition of *Monilinia fructicola* colony growth by *Aureobasidium pullulans* strains grouped according to their geographical origin ((**A**): FVG Region: (**B**): Algerian Desert; (**C**): Alto Adige Region; (**D**): Sweden Coast; (**E**): Fusine’s Lake; (**F**): France Urban Centre). Colony diameters were measured after 5 days of incubation at 25 °C. Each value is the mean of three plates ± standard error. Different letters represent significant differences among the groups according to Tukey’s Test (α = 0.05).

*Aureobasidium pullulans* strains were also tested as potential BCAs by a dual-culture assay (Supplemental Material Figure S3) against *M. fructicola*. In particular, some strains, such as THM_1 (Algerian Desert), CPB_6 (Fusine’s Lake), Ger2 (FVG Region), THM_2 (Algerian Desert), and SP6Gram_1 (Sweden Coast), did not display any inhibition against the target pathogen. Conversely, the strains RB_7 (Algerian Desert), ACB_8, and ACB_10 (Alto Adige Region) inhibited the pathogen colony growth on average by more than 55% compared to the control (Figure 2). Figure 2 reports the percentage of inhibition of *M. fructicola* colony growth by all the *A. pullulans* strains, clustered in groups relative to the geographical origins. For each group, only a few strains showed significant biocontrol activity against the fungal pathogen. In particular, in Group 1, (FVG Region) (Figure 2A) the most active strain was *A. pullulans* Ger3, which inhibited the fungal pathogen by 48.9% with respect to the control. In Group 2 (Algerian Desert) (Figure 2B), strains RB_3, RB_7, and THM_17 displayed on average an inhibition of the pathogen colony diameter by 51.8%, 56.5%, and 53.9%. Conversely, the strain RB_8, which belonged to the same group, was not effective against *M. fructicola.* In Group 3 (Alto Adige Region) (Figure 2C), only two strains, ACB_10 and ACB_8, showed a high inhibitory effect against the pathogen by 70% and 62%, respectively. In Groups 4 (Sweden Coast) (Figure 2D) and 5 (Fusine’s Lake) (Figure 2E), strains S1R_2 and FSC_16, respectively, were the most active microorganisms, showing an inhibitory effect by 35% (SIR_2) and 54% (FSC_16). In Group 6 (France Urban Centre) (Figure 2F), all four tested strains reduced pathogen colony growth by 21% on average.

### 3.6. In Vivo Assay

**Figure 3:**
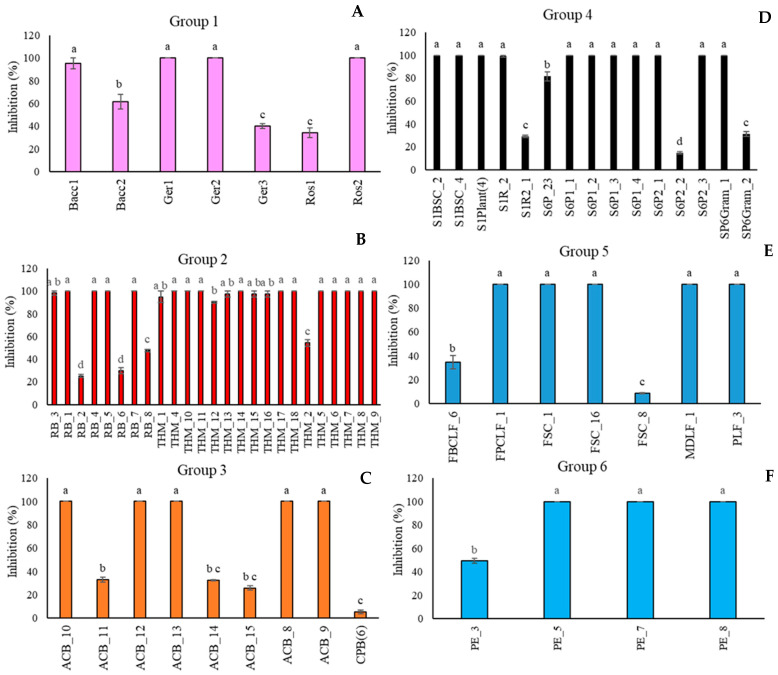
Percentage of inhibition of the disease severity of *Monilinia fructicola* on “Red Haven” peaches by *Aureobasidium pullulans* strains clustered in six groups on the basis of the geographical origins ((**A**): FVG Region; (**B**): Algerian Desert; (**C**): Alto Adige Region; (**D**): Sweden Coast; (**E**): Fusine’s Lake; (**F**): France Urban Centre). Each value is the mean of five fruits ± standard error. Different letters represent significant differences among the groups according to Tukey’s Test (α = 0.05).

In Figure 3, the biocontrol efficacy of *A. pullulans* strains was reported as the percentage of disease severity inhibition. For each group (Figure 3A–F), in contrast with the in vitro assay, most of the isolated strains showed significant biocontrol activity against the target pathogen, totally inhibiting its growth. Conversely to the in vitro results, the strain *A. pullulans* Ger3 (Group 1, FVG Region) (Figure 3A), which showed the highest percentage of inhibition against *M. fructicola* colony growth in the in vivo assay, resulted in the least control over brown rot on peaches. In Group 2 (Algerian Desert) (Figure 3B), the in vitro less active strain RB_8 also confirmed its performance in vivo.

### 3.7. Enzymatic Assays and Evaluation of Siderophore Production

The 12 *A. pullulans* strains, selected in relation to their different biocontrol potential activity, were tested to verify if their effectiveness was also related to the production of cellulase, xylanase enzymes, and siderophores (Table 6). All the strains produced cellulase and xylanase enzymes. No differences were detected between the strains in halo diameter production (on average 13.4 mm). Conversely, differences were detected for xylanase production; most of the strains displayed an enzymatic halo between 18 and 22 mm in diameter. Only the strain RB_7 (Algerian Desert) showed the largest enzymatic halo, between 26 and 28 mm.

### 3.8. Key Traits Driving Strain Differentiation in A. pullulans

To explore patterns in phenotypic responses across the 67 *A. pullulans* strains, a non-metric multidimensional scaling (NMDS) ordination was first conducted based on cell production, growth, and antagonism data under varying media, temperature, pH, and assay conditions. The NMDS yielded a two-dimensional solution with a stress value of 0.1907, indicating an acceptable level of dimensional reduction.

**Figure 4:**
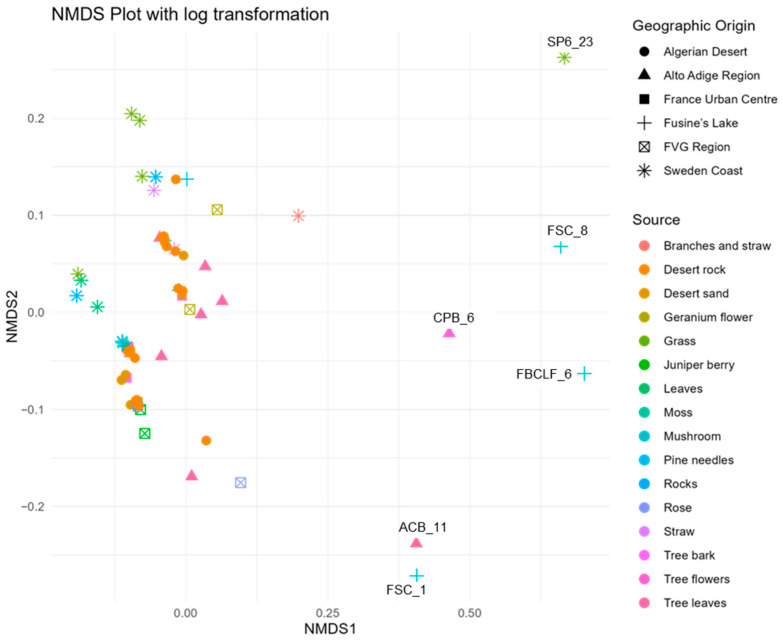
Non-metric multidimensional scaling (NMDS) illustrating the clustering patterns of *A. pullulans* strains isolated from diverse environmental sources, based on a multivariate analysis of phenotypic traits.

**Figure 5:**
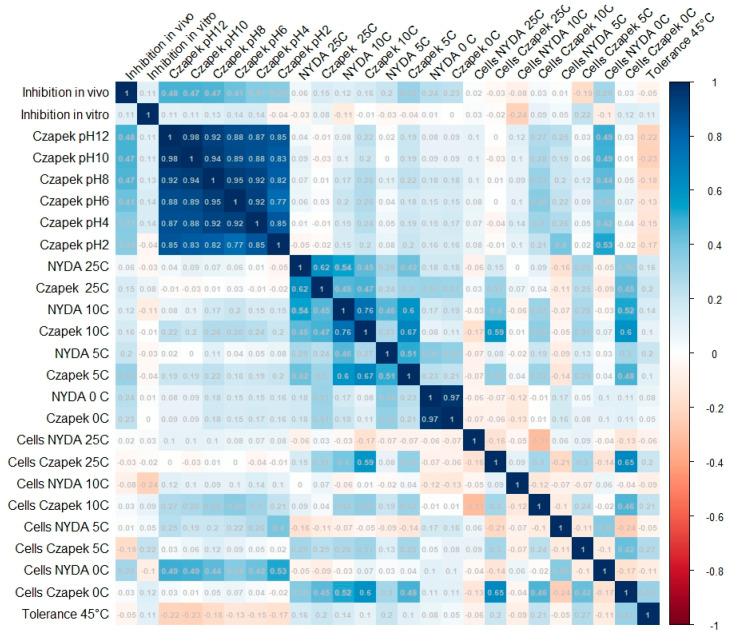
Complete correlation matrix between pairs of the tested cultural and biocontrol features of 67 *A. pullulans* strains.

**Figure 6:**
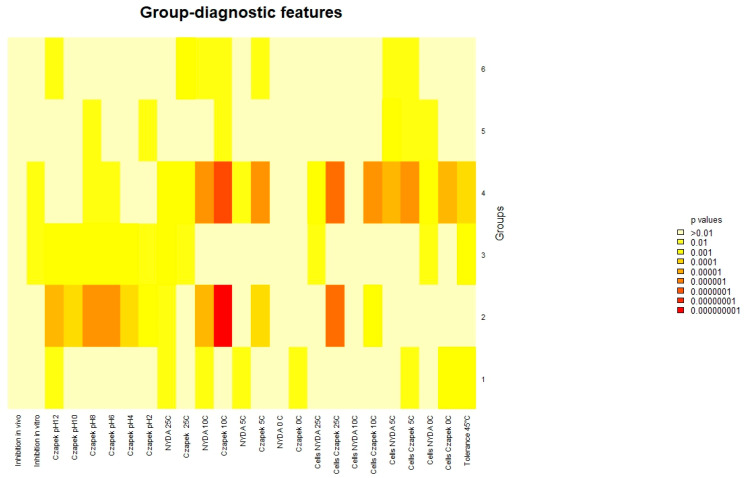
Wilcoxon test. Heatmap of the diagnostic power of the different growth and BCA efficacy for the prediction of the geographic origin.

NMDS revealed no strong or discrete clustering of strains by source or origin (Figure 4). Most strains were positioned relatively closely in ordination space, indicating similarity in their overall profiles. However, clear outliers emerged, marked by greater distances from the NMDS centroid. These outliers include FSC_8, S6P_23, FBCLF_6, FSC_1, CPB_6, and ACB_11, all positioned furthest from the centre of the NMDS space. All the outliers but S6P23 stand out for their poor growth at all the tested pH conditions on Czapek agar, while S6P23 showed formed diameter colonies at 5 °C and 10 °C. They did not noticeably differ from the in-group strains as far as the biological control activity was concerned. The outlier strains identified in the NMDS analysis showed relatively moderate and high antagonistic activity against *Monilinia*, with inhibition rates ranging from 28.5% (FSC_8) to 39.0% (S6P_23, ACB_11). Other outliers, such as CPB_6 (37.6%) and FBCLF_6 (37.9%), also showed strong inhibition. However, despite their high activity, these strains were not the most effective overall. The highest antagonistic effects were observed in non-outlier strains such as S1R2_1 (Sweden Coast, rocks, inhibition of 44.9%) and THM_13 (Algerian Desert, desert rock, inhibition of 44.2%), indicating that distinct NMDS positioning does not necessarily equate to superior antagonistic performance against *Monilinia.* Strain SP6_23 showed relatively high inhibition, but it did not show a good growth rate and did not produce cells on Czapek media at lower temperatures (0 °C), while FBCLF_6 and FSC_8 did not produce cells at 0 °C growing on NYDA, which is an important requirement for good biocontrol selection. In general, the biological control activities, as related to the grow restriction of *M. fructicola* in vivo or in vitro, could not be reliably predicted by any of the cultural tests performed in this work, as could be seen in the correlation matrix presented in Figure 5: For in vitro inhibition, low correlation values could only be detected with cell production on NYDA at 10 °C and on Czapeck at 5 °C. For in vivo inhibition, modest correlation could be established with colony diameter in all tests for growth at different pH values. Indeed, for each strain, the colony diameters measured for growth at pH 2, pH4, pH6, pH8, pH10, and pH12 were all relatively similar, as indicated by the high correlation values among the corresponding variables in the matrix of Figure 5. Despite the substantial overlap of the groupings based on the environment of origin clarified by the NMDS analysis, some of the assays used for strain differentiation among *A. pullulans* isolates provided results of interest, being the response typical of specific groups. Figure 6 shows a summary of the Wilcoxon tests carried out to compare the responses of the whole population analyzed with those of specific subpopulations. According to the results, the strains isolated from the Algerian Desert (Group 2) and those isolated from the Sweden Coast (Group 4) showed peculiar characteristics such as, for example, the diameter of the colony at 10 °C growing temperature on Czapeck agar, which was on average about 25 mm for Group 2 and 15 mm for Group 4. Other group diagnostic features can be detected in the heatmap of Figure 6 and can be further clarified by the box plots provided in Supplementary Material Figure S4.

## 4. Discussion

The present study provided evidence of *A. pullulans* strains’ environmental adaptability related to potential biocontrol efficacy. The molecular analysis of the strains confirmed the homogeneity of *A. pullulans*, as evidenced by Gostinčar et al. [38]. Only the strains belonging to Group 2 (Algerian Desert) were clustered separately from the other strains, perhaps due to a sparsely anthropized environment characterized by peculiar environmental factors. In general, however, phylogenetic analysis showed that, despite the large geographic distances, isolates from both warm and cold environments did not create clear and distinct clusters, conceivably because *A. pullulans* strains may be subjected to genetic recombination or share ancestral connections across populations [10].

In terms of ecological characteristics, the strains demonstrated impressive tolerance to a wide range of temperatures and pH values. Several fungal species exhibit remarkable adaptability to extreme environmental conditions, as we observed in *A. pullulans*. For instance, some fungi belonging to the *Aspergillus* genus demonstrated notable ecological plasticity, being capable of growing over a wide temperature range (4–40 °C) and pH values, including moderately alkaline conditions, which supports their ability to colonize different ecological niches [39], or their exceptional resilience, which allows them to survive in environments with low temperatures and pH values ranging from 1.5 to 9.8 [40]. In contrast, a few other *Aspergillus* species, although tolerant of temperature variations, display greater sensitivity to nutrient limitations, with marked reductions in growth observed on poor media [41]. These studies collectively support the hypothesis that fungi with high ecological flexibility, like *A. pullulans*, have evolved adaptive mechanisms that enable survival and metabolic activity across extreme and fluctuating environmental parameters. Such characteristics are particularly relevant in the context of climate change and biotechnological exploitation, where fungal strains with high plasticity represent an added value for industrial and environmental applications. The ability of *A. pullulans* strains to grow at low temperatures such as 0 °C is particularly notable. This suggests that these strains could benefit biocontrol applications in cooler or refrigerated environments [42]. Their adaptability to low temperatures is crucial for postharvest biocontrol, as the effectiveness of BCAs is often challenged by environmental conditions such as cold temperatures.

Additionally, the pH tolerance assay showed that most of *A. pullulans* strains can thrive across a wide pH range, from 2 to 12. This remarkable capability to adapt to highly acidic and alkaline conditions aligns with findings from other microorganisms, such as *Wickerhamomyces anomalus* and *Rhodotorula* spp., described as exceptional pH-tolerant species [43], or *Debaryomyces hansenii*, able to survive and proliferate at high pH levels [44]. From a biotechnological perspective, the ability of these organisms to resist across a broad pH range makes them promising candidates for industrial processes that require robustness under acidic or alkaline conditions. Also, our findings indicated that our *A. pullulans* strains may offer significant advantages for biocontrol in a wide range of environmental settings, from cold temperatures to different pH values. These facts underscore their potential adaptability to different soil or water chemistries [21]. Some strains, particularly those from Group 3 (Alto Adige Region), showed a preference for acidic environments, while others, like those from Group 2 and Group 6, were more tolerant to alkaline conditions. The findings emphasize the broad ecological niche that these yeasts can occupy.

The antagonistic effectiveness of many of the strains isolated in this work confirmed *A. pullulans* as a good BCA [34,45,46]. In vitro assays showed that several strains, including RB_7, ACB_8, and Ger3 ACB_10 (belonging to Group 2, Group 3, and Group 1, respectively), inhibited *M. fructicola* colony growth by over 50% and up to 70%. Several biological control agents (BCAs) have been studied for their effectiveness against *Monilinia* spp., including yeasts, bacteria, and filamentous fungi, showing promising results both in vitro and in vivo. Strains of *A. pullulans*, specifically L1 and L8, were found to completely inhibit *M. laxa* and *M. fructicola* while reducing *M. fructigena* infection by more than 89%, particularly at concentrations of 10^8^ CFU/mL [34].

In our study, in the in vivo assay the strain RB_7 (Group 2) confirmed its pronounced antagonistic ability in reducing *M. fucticola* severity, achieving complete inhibition (100%) of the pathogen. Interestingly, no clear correlation was found between the antifungal properties and the enzymatic activities of the tested *A. pullulans* strains. The mechanisms behind biocontrol activity probably involve other factors beyond enzyme production, such as competition for nutrients, antifungal secondary metabolites, or the induction of host plant defences. Furthermore, none of the tested strains produced siderophores under the conditions used in the study, indicating that iron chelation might not be a significant factor in their metabolic activity.

The NMDS analysis showed most of the strains clustered closely, indicating general similarity across sources and geographic origins. This result reflects a common pattern in microbial ecology where microbial populations often exist as a continuum rather than distinct groups, likely due to overlapping ecological niches and dispersal mechanisms [47]. This suggests that many strains share broadly similar ecological functions despite originating from diverse habitats. The distinct outliers identified are characterized by their poor growth performances in specific conditions, likely depending on function loss connected with specialized ecological roles or adaptations to unique substrates (e.g., mushrooms and tree flowers).

Despite the overlap in the NMDS space, some groups, such as Group 2 (Algerian Desert) and Group 4 (Sweden Coast), presented distinctive features that, although not affecting their overall similarity with other strains, typically characterize them. In particular, with reference to the whole population of 67 isolates, on the nutrient-poor substrate (Czapeck agar), the strains from Group 2 developed the largest colonies and produced cells most abundantly, while conversely, Group 4 developed the smallest colonies with the least-abundant cell production. It is therefore possible that a separation of the groups based on their environmental origin, which did not result from the NMDS analysis presented here, could be achieved by focusing and limiting the assay panel to the features relevant to extreme nutritional and environmental conditions. Also, the analyzed strains showed varying capacities to produce CWDEs; however, none of these produced siderophores under the tested conditions.

Obtained results showed that extreme environments, such as deserts and coastal regions, could represent good sources of strains with potential biocontrol ability. This fact highlights the crucial role of the environment when evaluating microbial strains for biocontrol purposes and for their functional traits [34]. However, biocontrol potential cannot be reliably predicted by the growth and sporulation performances of the strain on different substrates, pH, or temperature conditions. Nevertheless, some of these features may be distinctive of the environment of origin. Our findings underscore the ecological plasticity of *A. pullulans* and support its potential as a sustainable, climate-resilient BCA. These results displayed that strains of *A. pullulans* belonging to extreme environments could be promising microorganisms for future biological control strategy development.

## 5. Conclusions

Understanding the intricate biology of extremophilic microorganisms could be of considerable interest when it comes to evaluating their potential benefits, particularly with regard to plant health. One of the main objectives for the future is to investigate the real connections between places of isolation and biocontrol potential in order to identify sites and environments that are most likely to host extremophilic microorganisms that are also excellent BCAs.

However, further genomic-level analyses will be required to effectively assess these microorganisms’ predisposition to acting as effective antagonists, particularly through an in-depth analysis of their secondary metabolism. Investigating the biology of these microorganisms in depth is crucial for developing a sustainable agricultural strategy in the face of environmental fluctuations.

## Figures and Tables

**Table 1:** Yeast strains selected based on a different range of inhibition (%) against *Monilinia fructicola*.

Range of Inhibition (%)	Selected Strains
>45	RB_7
	ACB_8
	ACB_10
>30–35<	S6P_23
	THM_8
	THM_9
	FPCLF_1
	THM_12
	PE_3
25	S1BSC_2
	THM_13
	CPB_6

**Table 2:** *Aureobasidium pullulans* strains. ID codes, geographical origin, isolation sources, date of isolation, grouping, and coordinates.

Geographic Origin	Sample ID	Molecular Identification	Source	Date of Isolation	Group	Coordinates
FVG Region	Ger1	*A. pullulans*	Geranium flower	December 2023	1	46.081623° N, 13.211754° E Udine, 33100 UD, Italy
	Ger2	*A. pullulans*	Geranium flower			
	Ger3	*A. pullulans*	Geranium flower			
	Bacc1	*A. pullulans*	Juniper berry			
	Bacc2	*A. pullulans*	Juniper berry			
	Ros1	*A. pullulans*	Rose			
	Ros2	*A. pullulans*	Rose			
Algerian Desert	RB_1	*A. pullulans*	Desert sand	February 2024	2	31.190896° N, 5.548179° E Hassi Messaoud, Algeria
	RB_2	*A. pullulans*	Desert sand			
	RB_3	*A. pullulans*	Desert sand			
	RB_4	*A. pullulans*	Desert sand			
	RB_5	*A. pullulans*	Desert sand			
	RB_6	*A. pullulans*	Desert sand			
	RB_7	*A. pullulans*	Desert sand			
	RB_8	*A. pullulans*	Desert sand			
	THM_1	*A. pullulans*	Desert rock			
	THM_2	*A. pullulans*	Desert rock			
	THM_4	*A. pullulans*	Desert rock			
	THM_5	*A. pullulans*	Desert rock			
	THM_6	*A. pullulans*	Desert rock			
	THM_7	*A. pullulans*	Desert rock			
	THM_8	*A. pullulans*	Desert rock			
	THM_9	*A. pullulans*	Desert rock			
	THM_10	*A. pullulans*	Desert rock			
	THM_11	*A. pullulans*	Desert rock			
	THM_12	*A. pullulans*	Desert rock			
	THM_13	*A. pullulans*	Desert rock			
	THM_14	*A. pullulans*	Desert rock			
	THM_15	*A. pullulans*	Desert rock			
	THM_16	*A. pullulans*	Desert rock			
	THM_17	*A. pullulans*	Desert rock			
	THM_18	*A. pullulans*	Desert rock			
Alto Adige Region	ACB_8	*A. pullulans*	Tree leaves	March 2024	3	46.488848° N, 11.350958° E Bolzano, 39100 BZ, Italy
	ACB_9	*A. pullulans*	Tree leaves			
	ACB_10	*A. pullulans*	Tree leaves			
	ACB_11	*A. pullulans*	Tree leaves			
	ACB_12	*A. pullulans*	Tree leaves			
	ACB_13	*A. pullulans*	Tree leaves			
	ACB_14	*A. pullulans*	Tree leaves			
	ACB_15	*A. pullulans*	Tree leaves			
	CPB_6	*A. pullulans*	Tree flowers			
Sweden Coast	S1BSC_2	*A. pullulans*	Moss	January 2024	4	55.678328° N, 13.059162° E Lomma Beach, Sweden
	S1BSC_4	*A. pullulans*	Rock			
	S1Plant(4)	*A. pullulans*	Branches and straw			
	S1R_2	*A. pullulans*	Rocks			
	S1R2_1	*A. pullulans*	Rocks			
	S6P1_1	*A. pullulans*	Leaves			
	S6P1_2	*A. pullulans*	Leaves			
	S6P1_3	*A. pullulans*	Leaves			
	S6P1_4	*A. pullulans*	Grass			
	S6P2_1	*A. pullulans*	Grass			
	S6P2_2	*A. pullulans*	Grass			
	S6P2_3	*A. pullulans*	Grass			
	S6P_23	*A. pullulans*	Grass			
	SP6Gram_1	*A. pullulans*	Straw			
	SP6Gram_2	*A. pullulans*	Straw			
Fusine’s Lake	FPCLF_1	*A. pullulans*	Mushroom	February 2024	5	46.476432° N, 13.670997° E Tarvisio, 33018 UD, Italy
	FBCLF_6	*A. pullulans*	Mushroom			
	FSC_16	*A. pullulans*	Mushroom			
	MDLF_1	*A. pullulans*	Mushroom			
	PLF_3	*A. pullulans*	Pine needles			
	FSC_1	*A. pullulans*	Mushroom			
	FSC_8	*A. pullulans*	Mushroom			
France Urban Centre	PE_3	*A. pullulans*	Tree bark		6	48.848889° N, 2.337341° E Odéon, 75006 Paris, France
	PE_5	*A. pullulans*	Tree bark			
	PE_7	*A. pullulans*	Tree bark			
	PE_8	*A. pullulans*	Tree bark			

**Table 3:** Averaged growth diameter (mm) of the *Aureobasidium pullulans* strains included in six groups based on each geographical origin of isolation. The effect of agar medium (NYDA and Czapeck) and temperature (0 °C, 5 °C, 10 °C, 25 °C) on colony growth was considered. Different letters mean significant differences between the groups at the same temperature and medium.

Group	Colony Diameter (mm)							
	0 °C		5 °C		10 °C		25 °C	
	NYDA	CZAPEK	NYDA	CZAPEK	NYDA	CZAPEK	NYDA	CZAPEK
1	5.14 ± 0.73 a	5.57 ± 0.79 a	11.59 ± 0.39 a	11.80 ± 0.42 a	20.57 ± 0.32 a b	21.76 ± 0.63 b	25.21 ± 0.16 a	25.14 ± 0.18 a b
2	3.73 ± 0.41 a	3.93 ± 0.44 a	11.00 ± 0.33 a b *	13.00 ± 0.25 a b *	21.21 ± 0.19 a *	24.92 ± 0.15 a *	24.82 ± 0.28 a	24.30 ± 0.34 b
3	3.24 ± 0.71 a	2.46 ± 0.68 a	10.53 ± 0.44 a b	10.50 ± 0.46 b c	18.12 ± 0.65 c *	20.01 ± 0.48 b *	22.61 ± 0.37 b	21.92 ± 0.67 c
4	3.33 ± 0.53 a	3.41 ± 0.55 a	9.70 ± 0.36 b	8.82 ± 0.33 c	15.98 ± 0.52 d	15.26 ± 0.60 c	22.03 ± 0.39 b	21.66 ± 0.48 c
5	2.04 ± 0.72 a	2.23 ± 0.79 a	11.00 ± 0.32 a b	11.52 ± 0.36 a b	19.78 ± 0.62 b c	20.35 ± 0.65 b	24.97 ± 0.31 a	25.45 ± 0.32 a b
6	5.58 ± 0.98 a	5.50 ± 0.96 a	11.70 ± 0.64 a b	14.29 ± 0.59 a b c	21.04 ± 0.52 a *	24.00 ± 0.59 a *	23.87 ± 0.52 a b *	27.25 ± 0.33 a *

The asterisk (*) means significant statistical difference for the same group grown at the same temperature on different media, according to Tukey’s Test (α = 0.05).

**Table 4:** Average cell concentration (cells/mL) of *Aureobasidium pullulans* strains included in six groups based on each geographical origin of isolation. The effect of agar medium (NYDA and Czapeck) and temperature (0 °C, 5 °C, 10 °C, 25 °C) on cell production was considered. Different letters mean significant differences between the groups at the same temperature and medium.

Group	Conidia/mL							
	0 °C		5 °C		10 °C		25 °C	
	NYDA	CZAPEK	NYDA	CZAPEK	NYDA	CZAPEK	NYDA	CZAPEK
1	3.44 × 10^7^ c *	1.97 × 10^7^ a *	1.44 × 10^7^ b *	3.04 × 10^7^ a *	1.21 × 10^8^ a	2.57 × 10^7^ a b *	6.95 × 10^7^ a b *	4.63 × 10^6^ b *
2	7.97 × 10^7^ b *	3.25 × 10^6^ b *	3.79 × 10^7^ b	9.64 × 10^7^ a *	9.80 × 10^7^ a	2.54 × 10^7^ a *	8.61 × 10^7^ a b *	2.19 × 10^7^ a *
3	2.81 × 10^7^ c *	6.42 × 10^6^ b *	2.69 × 10^7^ b *	1.73 × 10^7^ a *	9.30 × 10^7^ a	2.03 × 10^7^ a b *	4.32 × 10^7^ b*	5.24 × 10^6^ b *
4	1.22 × 10^8^ a	6.00 × 10^5^ b *	1.01 × 10^8^ a	2.86 × 10^6^ a *	1.25 × 10^8^ a	2.03 × 10^6^ c *	1.34 × 10^8^ a	1.99 × 10^6^ b *
5	2.41 × 10^7^ c *	1.70 × 10^7^ a *	8.95 × 10^6^ b *	2.82 × 10^7^ a *	1.06 × 10^8^ a	3.54 × 10^6^ b c *	8.07 × 10^7^ a b	1.27 × 10^7^ a b *
6	1.08 × 10^8^ a b *	8.13 × 10^5^ b *	9.50 × 10^7^ a *	6.27 × 10^7^ a *	9.65 × 10^7^ a *	3.43 × 10^7^ a *	1.13 × 10^8^ a b	1.12 × 10^7^ a b *

The asterisk (*) means significant statistical difference within the same group at the same temperature grown on different media according to Tukey’s Test (α = 0.05).

**Table 5:** Average growth diameter (mm) of *Aureobasidium pullulans* strains included in six groups based on each geographical origin of isolation. The effect of pH values ranging from 12 to 2 on colony growth was considered. Different letters mean significant differences between the groups at the same pH value.

**Group**	**Colony Diameter (mm)**					
	**pH 12**	**pH 10**	**pH 8**	**pH 6**	**pH 4**	**pH 2**
1	18.88 ± 1.96 c d *	19.83 ± 2.04 b c d e *	23.33 ± 1.51 b c *	27.00 ± 0.42 a	26.76 ± 0.44 a b	18.52 ± 2.04 a b *
2	26.98 ± 0.46 a	26.94 ± 0.39 a	27.83 ± 0.33 a	28.86 ± 0.21 a	27.11 ± 0.31 a	22.86 ± 0.73 a *
3	16.65 ± 1.96 c d *	17.30 ± 1.95 d e *	22.65 ± 0.63 c	23.83 ± 0.45 b	16.51 ± 2.14 c d *	12.51 ± 2.21 b *
4	22.03 ± 1.17 b c	22.22 ± 1.16 c d	23.55 ± 0.85 c d	24.33 ± 0.82 b	21.42 ± 1.41 b c	19.34 ± 1.29 a
5	15.64 ± 3.07 d *	15.62 ± 3.06 e *	25.95 ± 0.51 e *	26.95 ± 0.61 a	14.98 ± 2.93 d *	12.76 ± 2.71 b *
6	28.63 ± 0.22 a b	27.96 ± 0.21 a b c *	27.00 ± 0.75 a b c *	26.87 ± 0.96 a b *	26.13 ± 0.69 a b *	24.61 ± 0.53 a *

The asterisk (*) means significant statistical difference between the different pH within the same group according to Tukey’s Test (α = 0.05).

**Table 6:** Enzymatic activity and siderophore production by twelve *Aureobasidium pullulans* strains.

Strain	Cellulase	Xylanase	Siderofore	Group
RB_7	+	++++	-	2 Group
THM_12	+	++	-	
THM_9	+	++	-	
THM_13	+	++	-	
THM_8	+	+++	-	
ACB_8	+	++	-	3 Group
CPB_6	+	++	-	
ACB_10	+	+++	-	
S1BSC_2	+	++	-	4 Group
S6P_23	+	++	-	
FPCLF_1	+	+++	-	5 Group
PE_3	+	++	-	6 Group

+: 11–15 mm; ++: 18–21 mm; +++: 22–26 mm; ++++: 26–28 mm; -: no production.

## Data Availability

The original contributions presented in this study are included in the article/Supplementary Material. Further inquiries can be directed to the corresponding author.

## Supplementary Materials

The following supporting information can be downloaded at: https://www.mdpi.com/article/10.3390/microorganisms13112596/s1, Figure S1: Sampling origin: (A) Sweden Coast, (B) Alto Adige Region, (C) Fusine’s Lake, (D) Algerian Desert, (E) France Urban Centre, (F) Friuli Venezia Giulia Region (FVG Region); Figure S2: *Aureobasidium pullulans* strains growth at 2 pH, 4 pH, 6 pH, 8 pH, 10 pH, and 12 pH. Strains were included in six groups based on each geographical origin of isolation; Figure S3: Dual culture of the selected strains belonging to different groups and geographical origin against *Monilinia fructicola* strain AMF1; Figure S4: Dot plot analysis of the most significant variables relating to the outliers based on the NMDS graph.
