# Supplementary material for: Strains of Aureobasidium pullulans from Extreme Environments: New Potential Biocontrol Agents?
Source: Microorganisms. 2025 Nov 14;13(11):2596. doi: 10.3390/microorganisms13112596 (PMC12654642; doi:10.3390/microorganisms13112596)
Supplement: Supplementary file 1 [file microorganisms-13-02596-s001.zip › microorganisms-3942190-supplementary.pdf]

**Supplementary material**

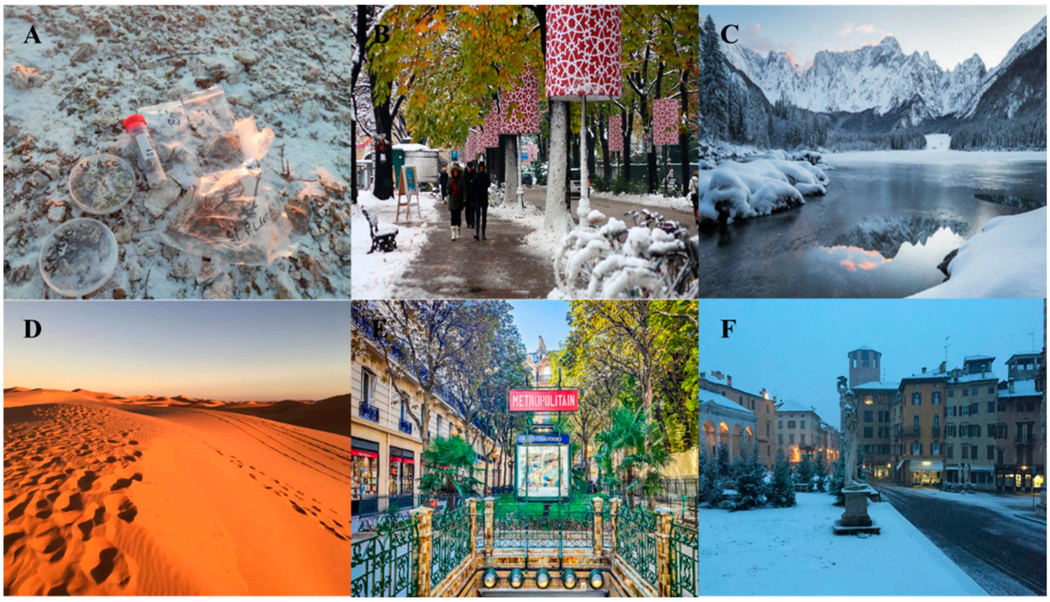


**Figure S1.** Sampling origin: (A) Sweden Coast, (B) Alto Adige Region, (C) Fusine's Lake, (D) Algerian Desert, (E) France Urban Centre (F) Friuli Venezia Giulia Region (FVG Region).

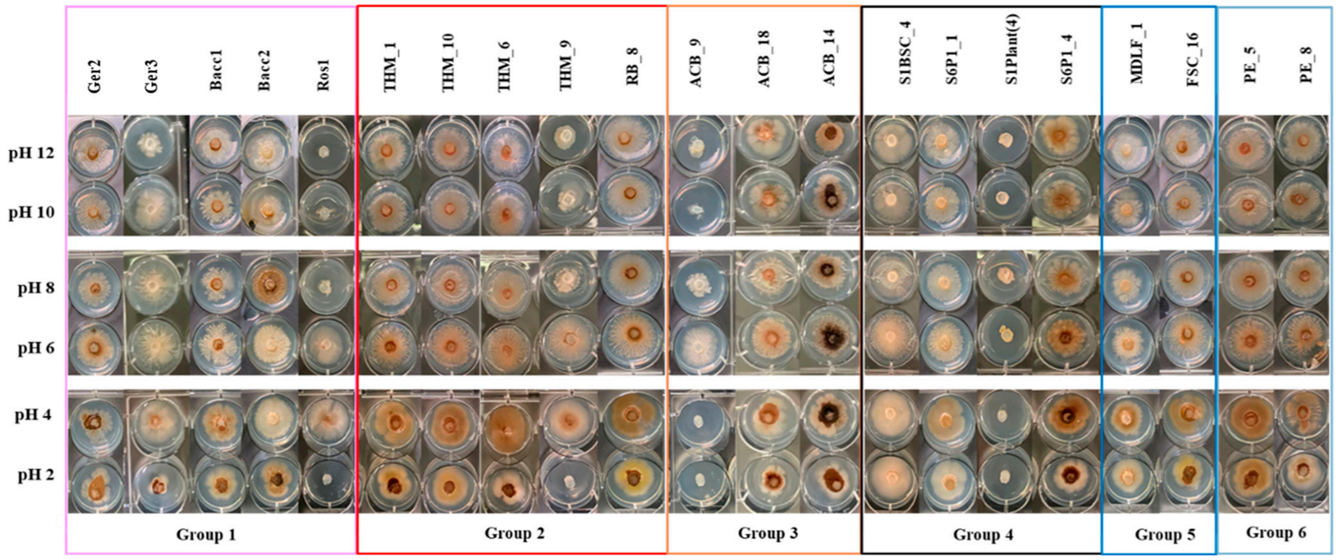


**Figure S2.** *Aureobasidium pullulans* strains growth at 2 pH, 4 pH, 6 pH, 8 pH, 10 pH, and 12 pH. Strains were included in 6 Groups based on each geographical origin of isolation.

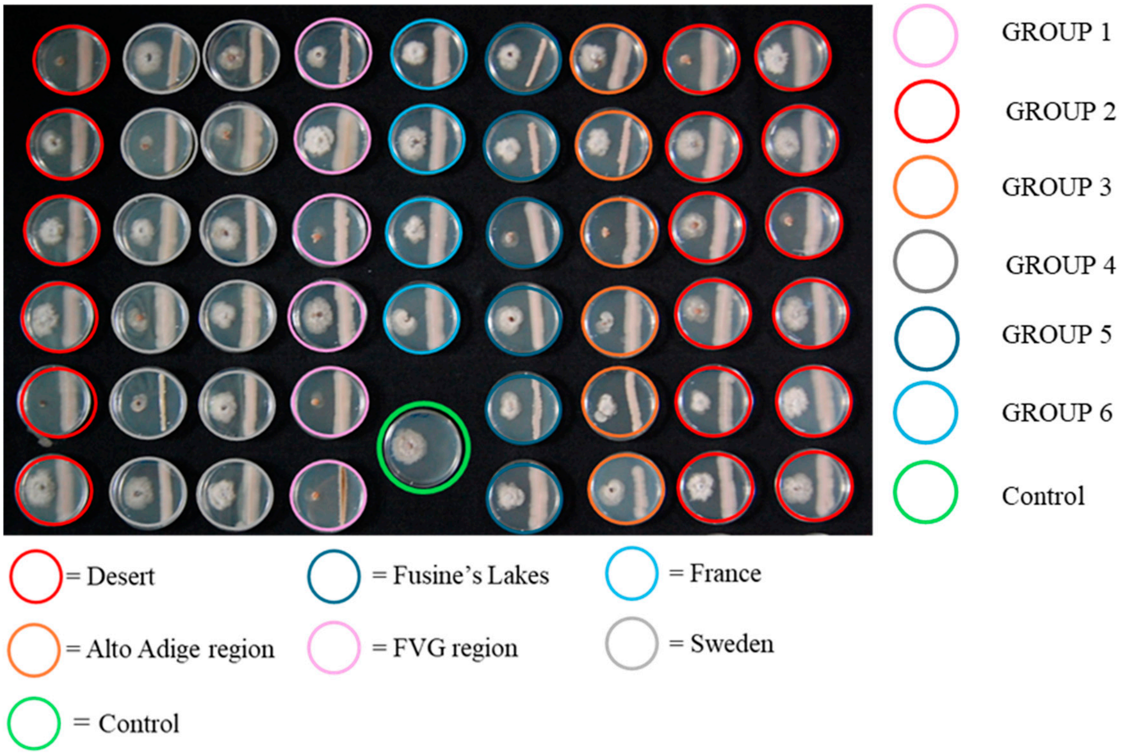


**Figure S3.** Dual culture of the selected strains belonging to different Groups and geographical origin against *Monilinia fructicola* strain AMF1.

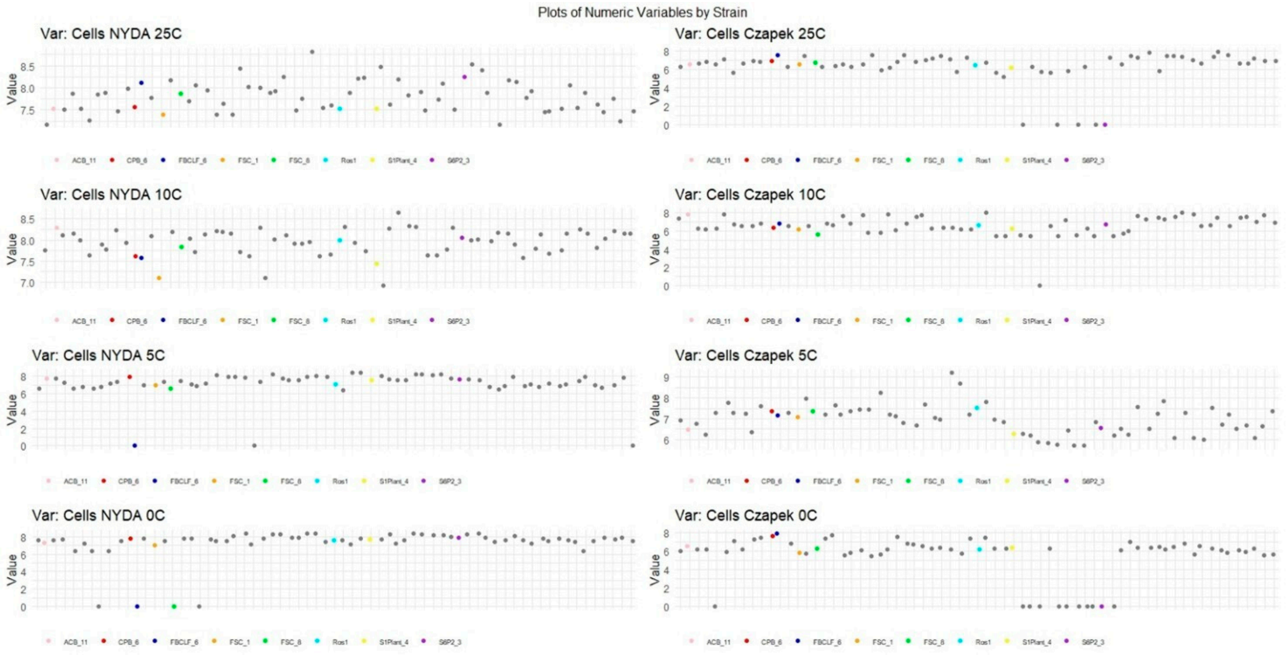


**Figure S4**. Dot Plot analysis of the most significant variables relating to the Outliers based on the NMDS graph.
